# Machine learning prediction of UV–Vis spectra features of organic compounds related to photoreactive potential

**DOI:** 10.1038/s41598-021-03070-9

**Published:** 2021-12-09

**Authors:** Rafael Mamede, Florbela Pereira, João Aires-de-Sousa

**Affiliations:** LAQV-REQUIMTE, Department of Chemistry, NOVA School of Science and Technology, Universidade Nova de Lisboa, 2829-516 Caparica, Portugal

**Keywords:** Drug safety, Cheminformatics, Photochemistry

## Abstract

Machine learning (ML) algorithms were explored for the classification of the UV–Vis absorption spectrum of organic molecules based on molecular descriptors and fingerprints generated from 2D chemical structures. Training and test data (~ 75 k molecules and associated UV–Vis data) were assembled from a database with lists of experimental absorption maxima. They were labeled with positive class (related to photoreactive potential) if an absorption maximum is reported in the range between 290 and 700 nm (UV/Vis) with molar extinction coefficient (MEC) above 1000 Lmol^−1^ cm^−1^, and as negative if no such a peak is in the list. Random forests were selected among several algorithms. The models were validated with two external test sets comprising 998 organic molecules, obtaining a global accuracy up to 0.89, sensitivity of 0.90 and specificity of 0.88. The ML output (UV–Vis spectrum class) was explored as a predictor of the 3T3 NRU phototoxicity in vitro assay for a set of 43 molecules. Comparable results were observed with the classification directly based on experimental UV–Vis data in the same format.

## Introduction

The UV–Vis absorption spectrum is a key physical property of an organic compound that determines many of its optoelectronic properties and photochemical reactivity. In the human body, the combined effect of an external chemical compound (e.g., plant toxins, phytomedicines, cosmetics, agrochemicals, food additives, dyes, drugs) and exposure to light, especially ultraviolet and visible radiation may give rise to an acute unwanted response in the skin or retina, which is called chemical phototoxicity^1,2^.

The prediction of UV–Vis spectra from the molecular structural formula is of general high interest to design new materials, identify potential phototoxic compounds, estimate missing spectroscopic data for known molecules, or curate databases of experimental spectra.

Machine learning (ML) techniques have been reported for the prediction of optical and photophysical properties of organic compounds^3–6^. Joung et al.^3^ reported a deep learning model developed with an experimental database of 30 ,094 chromophore/solvent combinations to predict several optical properties, namely, the first absorption peak position, bandwidth, and extinction coefficient, the emission peak position, bandwidth, and photoluminescence quantum yield; and illustrated the possibilities of applying ML to find target molecules with desired optical and photophysical properties. The root mean squared errors of the predicted values were found to be 26.6 and 28.0 nm for absorption and emission peak positions. A comparison between predictions of the absorption and emission spectra of coumarin 153 in ethanol using the ML model and TD-DFT calculations revealed a better performance of the ML model when compared to the theoretical calculations^3^. Another database of experimental and computational UV–Vis absorption spectra attributes was recently obtained through mining methods^7^.

ML algorithms can also be trained with theoretically calculated data sets obtained, e.g., by DFT methods, for faster estimation of molecular properties^8–11^. ML models based on theoretical optical spectra pre-calculated by finite-difference time-domain (FDTD) simulations for gold nanoparticles and nanorods were reported by Pashkov et al.^4^ The models were explored both to predict structural parameters for a given spectrum and to predict a spectrum for given structural parameters. Gosh et al.^5^ calculated a database of 132 k excitation spectra using the PBE density functional augmented with vdW corrections, and trained neural networks with various architectures to predict the spectra from the 3D structure. Kang et al.^6^ used random forests to predict the highest oscillator strength and associated excitation energy among ten excitation states of molecules from 1 and 2D descriptors of the molecule. The model was trained with the TD-DFT results of about half a million molecules.

Phototoxicity is strongly related to molecular photochemistry and photostability^2^. The optimization of ADME-Tox parameters (absorption, distribution, metabolism, excretion, and toxicology) using high-throughput tools is of great importance in drug discovery^12^, and ML approaches can be used to rationalize and predict phototoxicity, representing a valuable strategy for reducing experimental tests, if an acceptable level of accuracy of the developed models is ensured. Although several efforts have been reported to model phototoxicity directly from molecular structures^13–17^, the inclusion of spectroscopic information can improve predictive models^2^ and add chemically sound indicators that can be theoretically calculated or learned from more easily available and larger data sets.

Training ML models to predict full UV–Vis spectra requires large databases of spectra obtained under consistent conditions to predict multiple continuous variables (e.g., the molar extinction coefficients at several wavelengths). Differently, here we report the exploration of ML tools to *classify* organic molecules in terms of their UV–Vis absorption spectrum based on molecular descriptors.

The relationship between features of the UV–Vis absorption spectrum (“photoactivity”) and phototoxicity can be clearly understood from the ICH S10 guidance on photosafety evaluation of pharmaceuticals^18^, according to which a molecule is potentially photoreactive if it absorbs light in the range between 290 and 700 nm (UV/Vis) with molar extinction coefficient (MEC) greater than 1000 L∙mol^−1^∙cm^−1^. Excitation of molecules by light can lead to generation of reactive oxygen species and this can be an indicator of phototoxicity potential^18^. If the substance does not have a MEC above 1000 L∙mol^−1^∙cm^−1^ in the above-mentioned window no direct phototoxicity is anticipated in humans^18^.

We retrieved data from the Reaxys^®^ (https://www.reaxys.com) database^19^ for > 80,000 molecules, and positive/negative classes related to photoreactive potential were assigned from the lists of absorption maxima and molar extinction coefficients with threshold values based on the ICH S10 guidance.

An external data set of molecules for which data was available both for UV–Vis absorption^19^ and for in vitro phototoxicity assays^2^ was used to evaluate the overlap of correlations between a phototoxicity test and the UV–Vis spectrum class (experimental or predicted by ML). However, we would like to emphasize that this study aimed at training ML models to predict *features of the UV–Vis spectra* from the molecular structure, rather than predicting phototoxicity or evaluating the usefulness of spectroscopic data to predict phototoxicity.

## Methods

### Data sets/selection of training and test sets

Molecular structures were retrieved from the Reaxys^®^ database (https://www.reaxys.com)^19^ with associated UV–Vis absorption maxima and molar extinction coefficient (MEC) values and were filtered for molecular weight in the range 98–1080 g/mol, only one fragment, methanol as the solvent, exclusion of molecules with metal atoms, and restriction to publication date before 2016. The molecular structures were standardized by normalizing tautomerism, mesomerism and aromaticity using the Standardizer program version 19.19.0, ChemAxon (https://www.chemaxon.com). Duplicates were removed based on InChI identifiers and stereochemistry was not considered so that stereoisomers were considered as duplicates. Compounds with a non-zero global charge, radicals or valence errors were also discarded. The final data set comprises 74,784 molecules: 37,038 molecules assigned to the positive class (POS, molecules with one or more absorption maxima between 290–700 nm with MEC ≥ 1000 Lmol^−1^ cm^−1^) and the remaining 37,746 molecules assigned to the negative class (NEG). The definition of the classes was based on the ICH S10 guidance on photosafety evaluation of pharmaceuticals^18^.

The data set was randomly divided into a training set of 72,788 molecules (POS class: 36,036 molecules and NEG class: 36,752 molecules), a test set I of 998 molecules (POS class: 501 molecules and NEG class: 497 molecules), and a test set II of 998 molecules (POS class: 512 molecules and NEG class: 486 molecules). The test set II includes 43 molecules for which the result of the 3T3 NRU phototoxicity in vitro assay is also available from Schmidt et al.^2^ Table 1 shows the distribution of UV–Vis absorption features in the data sets.

**Table 1:** Distribution of UV–Vis absorption features in the data sets (MEC values in Lmol^−1^ cm^−1^).

	Training set	Test set I	Test set II
**POS class**^a^			
1000 ≤ MEC ≤ 5000	21.3	22.4	19.5
5000 ≤ MEC < 10,000	24.0	23.3	25
MEC ≥ 10,000	54.7	54.3	55.5
**NEG class**^b^			
λ < 290 nm, MEC < 1000	10.4	10.7	10.5
λ < 290 nm, MEC ≥ 1000	91.1	88.9	91.6
λ > 700 nm, MEC < 1000	0.005	0	0
λ > 700 nm, MEC ≥ 1000	0.07	0.20	0
290 ≤ λ ≤ 700 nm, MEC ≤ 900	6.5	8.0	5.6
290 ≤ λ ≤ 700 nm, MEC > 900	0.23	0.4	0.21

^a^Statistics concerning the peak with the highest MEC within the 290–700 nm window; ^b^ statistics concerning any listed peak.

### Calculation of molecular descriptors and fingerprints

Molecular fingerprints and 1D&2D molecular descriptors were calculated with PaDEL-Descriptor version 2.21^20^, and RDKit^21^. Different types of fingerprints with different sizes were calculated and explored: 166 MACCS (MACCS keys), 307 Substructure (presence and count of SMARTS patterns for Laggner functional group classification—Sub and SubC respectively), 881 PubChem fingerprints^22^, 1024 CDK (circular fingerprints), 1024 CDK extended (circular fingerprints with additional bits describing ring features), and 1024 MorganFP^23^. The 1D&2D molecular descriptors comprise 1443 descriptors, including electronic, topological, and constitutional descriptors.

Modified Distance Descriptors (Md)^10^ are based on the molecular connectivity thus making no use of bond orders and atomic formal charges (avoiding the generation of a 3D conformer, the application of an aromaticity definition and the mesomerism standardization). The descriptors count the pairs of atoms in a molecule at specific “modified distances”. Modified distances were defined in terms of the van der Waals radius of the atoms and Sanderson electronegativity of neighbors. Md descriptors were calculated for 1010 intervals, using a resolution of 0.017, interatomic distances up to 4 bonds, and a distance factor of 4.

Estimated molecular orbital energies (*E*_HOMO_, *E*_LUMO_ and GAP) were obtained with previously in-house developed ML models trained with DFT calculated data^10^—ML quantum descriptors (ML_QD_). They include 10 values obtained for the three properties with different models.

The calculators for some types of descriptors/fingerprints did not process all molecules, and the corresponding training sets have slightly different sizes: 72,787 for RDKit and RDKit Morgan fingerprints; 72,771 for MACCS, Sub, SubC and PubChem fingerprints; 72,747 for 1D&2D molecular descriptors; 72,770 for ML_QD_ descriptors.

### Selection of descriptors

In the quest for QSPR models with reduced number of descriptors, descriptor selection was performed based on the importance of descriptors assessed by RF (mean decrease in accuracy measure) with the R program version 3.6.1.^24^.

### Machine learning (ML) methods

Classification and Regression Trees (CART)^25^ operate by recursive partitioning of the initial data set with the goal of maximizing an information gain function (or variance reduction in regression trees) calculated for the various branches and terminal nodes. The best tree size is identified among sub-trees by cross-validation, or splits are not attempted if improvements above a threshold are not attained. Classification trees were built using the rpart package^26^ of the R program version 3.6.1^24^ with default parameter values, except for 1D&2D descriptors (the cp parameter was set at 0.05).

Random forests (RF)^27^ were implemented as ensembles of unpruned classification trees which are grown using bootstrap samples of the training set. Each individual tree is different because bootstrap samples vary, and randomly selected subsets of descriptors are made available for each node split. Predictions are obtained by a majority vote of the classification trees in the forest. An internal cross-validation error (or out-of-bag estimation, OOB) is directly calculated with the prediction error for the objects left out in the bootstrap procedure. The importance of a descriptor can be assessed by the mean decrease in accuracy when the values of the descriptor are randomly permuted. A probability is assigned to every prediction based on the number of votes obtained by the predicted class. RFs were grown with the R program^24^, version 3.6.1, using the randomForest library^28^. The model was manually optimized recurring to the OOB estimation, with the number of trees from 500 to 1000 and the number of available descriptors for each rule (mtry) equal to the square root or 1/3 of the total number of descriptors.

Support Vector Machines (SVM)^29^ map the training data into a hyperspace through a nonlinear mapping (a boundary or hyperplane) and then separate the classes of objects in this space. The boundary is positioned using examples in the training set—the support vectors. Kernel functions can be used to transform data into a hyperspace where the classes become linearly separable. In this study, SVM were implemented with the program Weka version 3.8.3^30^, using the LIBSVM package^31^. The type of SVM was set to C-SVM-classification and the radial basis function was used for the kernel function. Hyper-parameter tuning was performed with the *Experimenter* application in Weka using ten-fold cross-validation. C and γ values varied from 1 to 1000 and 0.003 to 0.0045, respectively. The C and γ values were finally set to 500 and 0.004, respectively, and the other parameters were used with default values.

Deep Learning Multilayer Perceptron Networks (_d_MLP) were trained and applied with the software library Keras^32^ version 2.2.5 based on Tensorflow numerical backend engine^33^. The feed-forward neural network architecture was manually optimized in terms of the number of hidden layers (2 to 6), weights initializer (random normal and Glorot uniform), optimizer (adadelta, adam, SGD, and SGD-nesterov), learning rate (0.0001 to 0.01) and decay (0 to 0.01). The final hyper-parameter settings were selected for our study based on a best of 10 validation experiments with the training set—Table 2.

**Table 2:** Hyper-parameter settings of the best _d_MLP model.

Hyper-parameter	Setting
Initializer	Random normal
Number of hidden layers	4
Number of neurons in the 1st and 2nd layers	250
Number of neurons in the 3rd layer	8
Number of neurons in the 4th layer	4
Number of neurons in the 5th layer	1
Activation 1st-4th layers	Relu
Activation 5th layer	Sigmoid
Batch size	36
Optimizer	Adam
Loss	Binary crossentropy
Epochs	100
Learning rate	0.001
Decay	10^–6^

### Statistical measures of models’ performance

Models were evaluated with external test sets and by internal validation with the training set. An OOB estimation for RF and a tenfold cross-validation for the other ML techniques procedures were employed with the training set. The following measures were calculated: true positives (TP), true negatives (TN), false positives (FP), false negatives (FN), sensitivity (SE), specificity (SP), overall predictive accuracy (Q) and Matthews correlation coefficient (MCC) were calculated with Eqs. (1)–(4).

1 $$SE = \frac{TP}{{TP + FN}}$$

2 $$SP = \frac{TN}{{TN + FP}}$$

3 $$Q = \frac{TP + TN}{{TP + FN + TN + FP}}$$

4 $$MCC = \frac{TPXTN - FNXFP}{{\sqrt {\left( {TP + FN} \right)\left( {TP + FP} \right)\left( {TN + FN} \right)\left( {TN + FP} \right)} }}$$

## Results and discussion

### Machine learning prediction of UV–Vis photoreactive potential

Several ways of representing the molecular structures were evaluated as input to RF classification models, which were trained to predict the UV–Vis spectrum class. The number of trees in the forest was set to 500, the number of descriptors available for each rule was the square root of the total number of descriptors and the other parameters were used with default values. The performance of the models was evaluated by internal validation with the training set (out-of-bag estimation, OOB) and by validation with test set I (998 molecules)—Table 3.

**Table 3:** Evaluation of different molecular descriptors and fingerprints for the prediction of UV–Vis spectrum class using the RF algorithm.

Descriptors	Q Tr^a^	Q^b^	SP^c^	SE^d^	MCC^e^	TP^f^	TN^g^	FP^h^	FN^i^
RDKitMorganFP	0.88	0.88	0.88	0.88	0.76	441	435	62	60
ExtCDK	0.89	0.87	0.86	0.9	0.75	448	425	72	53
CDK	0.89	0.87	0.85	0.9	0.74	449	421	76	52
MACCS	0.85	0.87	0.85	0.89	0.74	444	422	75	57
Md	0.87	0.87	0.85	0.89	0.74	448	422	75	53
PubChem	0.88	0.86	0.85	0.88	0.73	443	420	77	58
RDKitFP	0.87	0.87	0.85	0.88	0.73	442	425	73	58
1D&2D	0.86	0.85	0.83	0.87	0.7	434	415	83	66
SubC	0.85	0.84	0.81	0.86	0.67	430	404	93	71
Sub	0.8	0.8	0.77	0.83	0.61	420	382	115	81
ML_QD_	0.77	0.75	0.72	0.79	0.51	391	362	138	107

^a^Overall predictive accuracy for the training set in OOB estimation. ^b^Overall predictive accuracy (test set I). ^c^Specificity (test set I). ^d^Sensitivity (test set I). ^e^MCC, Matthews correlation coefficient (test set I). ^f^True Positives (test set I). ^g^True Negatives (test set I). ^h^False Positives (test set I). ^i^False Negatives (test set I).

The models with CDK, ExtCDK, and RDKitMorganFP showed the best overall predictive accuracy for the training set in the OOB estimation and similar predictions were achieved for the test set. Both CDK and ExtCDK representations yielded slightly higher sensitivity than RDKitMorganFP, but the latter enabled the highest specificity and MCC of all models. It is also worth mentioning that the few ML_QD_ descriptors alone provided results that, although worse, are still good.

The complementary potential of several molecular representations was investigated next by combining Md, ExtCDK, RDKitMorganFP, 1D&2D and ML_QD_ molecular descriptors/fingerprints. The criteria for generating the combinations were the complementary nature of the attributes and, in case of similar sets, those yielding better predictions individually. The results in Table 4 show that combined descriptors did not provide any significantly superior model.

**Table 4:** Evaluation of the performance of combined descriptors for the prediction of UV–Vis spectrum class using the RF algorithm.

Descriptors	Q Tr^a^	Q^b^	SP^c^	SE^d^	MCC^e^	TP^f^	TN^g^	FP^h^	FN^i^
ExtCDK + ML_QD_	0.89	0.88	0.86	0.90	0.76	452	428	69	49
ExtCDK + Md	0.89	0.88	0.86	0.89	0.75	446	429	68	53
RDKitMorganFP + Md	0.88	0.87	0.85	0.88	0.73	441	424	73	60
RDKitMorganFP + ML_QD_	0.87	0.86	0.84	0.88	0.73	443	418	79	58
1D&2D + ML_QD_	0.87	0.86	0.83	0.88	0.72	442	414	83	59
Md + ML_QD_	0.87	0.86	0.84	0.87	0.71	436	418	79	65
ExtCDK + 1D&2D	0.87	0.86	0.84	0.87	0.71	438	416	81	63
Md + 1D&2D	0.87	0.86	0.85	0.86	0.71	432	422	75	69
RDKitMorganFP + 1D&2D	0.86	0.85	0.83	0.86	0.70	434	413	84	68

^a^Overall predictive accuracy for the training set in OOB estimation. ^b^Overall predictive accuracy (test set I). ^c^Specificity (test set I). ^d^Sensitivity (test set I). ^e^MCC, Matthews correlation coefficient (test set I). ^f^True Positives (test set I). ^g^True Negatives (test set I). ^h^False Positives (test set I). ^i^False Negatives (test set I).

The impact of random fluctuations in the models was assessed by re-training with different seed initialization of random functions. Fluctuations of Q up to 2% were observed meaning that differences below this value cannot be considered significant. The best models were also validated with Y-scrambling experiments in which the percentage of correct predictions varied between 49.85% and 50.1% in 5 experiments.

**Figure 1:**
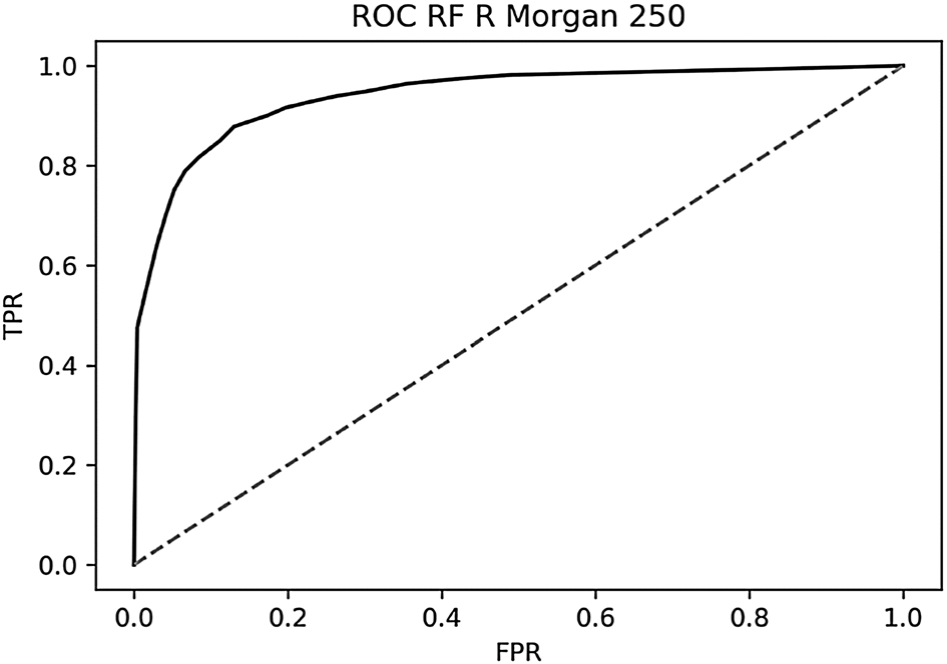
Receiver operating characteristic curve (ROC) obtained for the test set I with the RF model trained with 250 RDKitMorganFP attributes.

The RDKitMorganFP model achieved the best results for the test set with a Q of 0.88 and a MCC of 0.76 (Table 3). The 250 most important fingerprint bits of the RDKitMorganFP were identified by the RF model for the training set and were selected for training a new RF model with lower computational cost, as well as other models with different ML algorithms (SVM and _d_MLP)—Table 5. Reduction of attributes yielded a RF model with essentially the same quality. The receiver operating characteristic curve (ROC) obtained for the test set I with the RF model trained with 250 RDKitMorganFP attributes is displayed in Fig. 1. Superior results could not be observed with the alternative ML algorithms.

**Table 5:** Evaluation of alternative ML algorithms for the prediction of UV–Vis absorption spectrum class using 250 selected RDKitMorganFP molecular attributes.

Model	Q Tr^a^	Q^b^	SP^c^	SE^d^	MCC^e^	TP^f^	TN^g^	FP^h^	FN^i^
RF	0.88	0.87	0.87	0.88	0.75	440	431	66	61
_d_MLP	0.94	0.82	0.82	0.82	0.65	412	409	88	89
SVM	0.87	0.84	0.82	0.85	0.68	428	410	87	75

^a^Overall predictive accuracy for the training set in OOB estimation. ^b^Overall predictive accuracy (test set I). ^c^Specificity (test set I). ^d^Sensitivity (test set I). ^e^MCC, Matthews correlation coefficient (test set I). ^f^True Positives (test set I). ^g^True Negatives (test set I). ^h^False Positives (test set I). ^i^False Negatives (test set I).

The best model (RF 250 RDKitMorganFP) was further validated with the independent test set 2. The statistical parameters of the obtained predictions are in line with those obtained for the first test set. Models trained with CDK and ExtCDK fingerprints (default number of features) were also evaluated (Table 6).

**Table 6:** Evaluation of RF models trained with circular fingerprints to predict the UV–Vis absorption spectrum of organic molecules in test set II.

Model	Q^a^	SP^b^	SE^c^	MCC^d^	TP^e^	TN^f^	FP^g^	FN^h^
RDKitMorganFP	0.89	0.88	0.90	0.78	454	432	60	52
ExtCDK	0.89	0.87	0.91	0.78	460	428	64	46
CDK	0.88	0.86	0.90	0.77	458	423	68	49

^a^Overall predictive accuracy. ^b^Specificity. ^c^Sensitivity. ^d^MCC, Matthews correlation coefficient. ^e^True Positives. ^f^True Negatives. ^g^False Positives. ^h^False Negatives.

### Analysis of important molecular attributes

**Figure 2:**
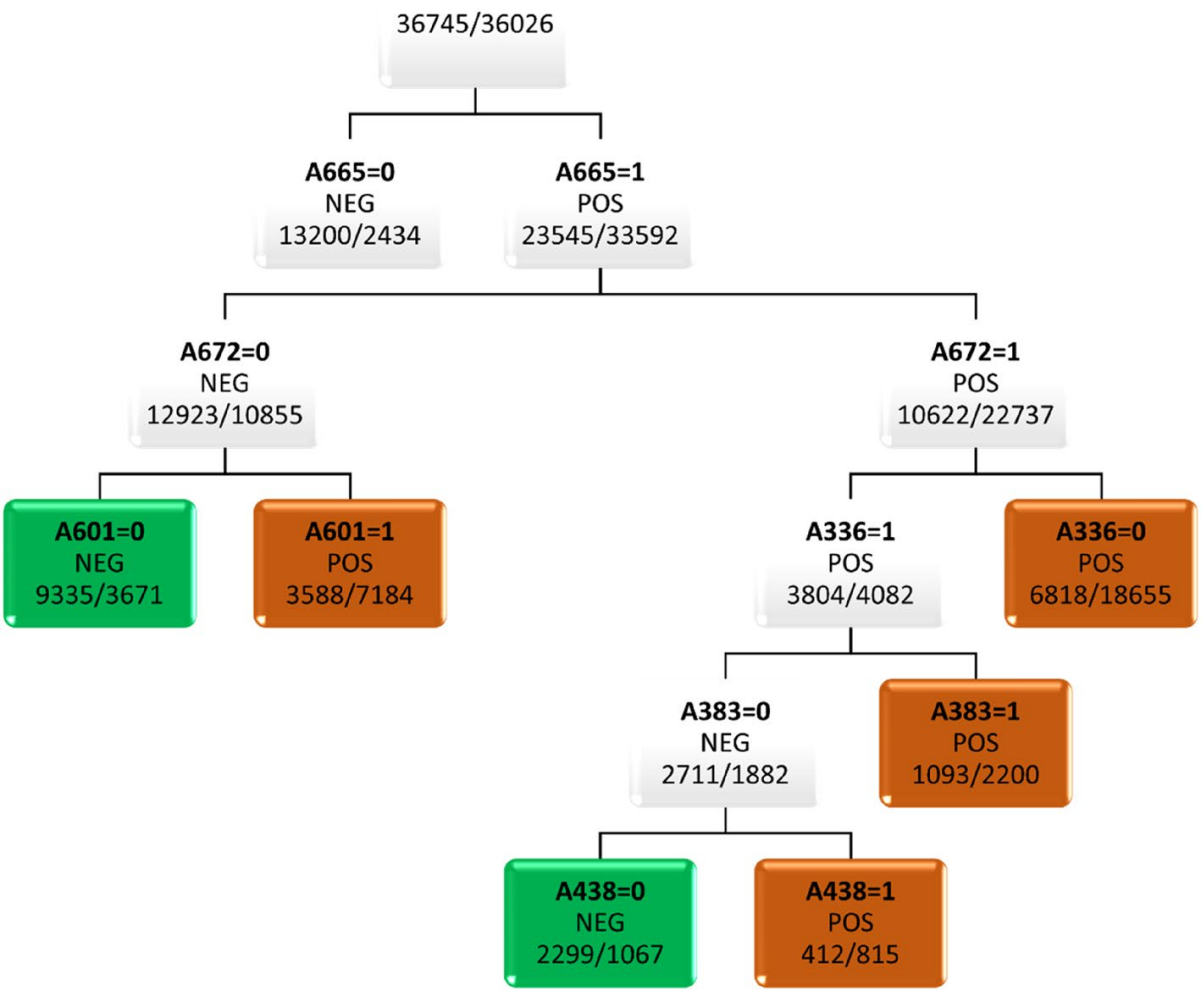
Classification tree based on PubChem fingerprints for the discrimination of molecules of the POS/NEG classes related to potential photoreactivity. A665, C–C=C–C=C; A672, O=C–C=C–C; A601, N–C:C:C–C; A336, C(~C)(~C)(~C)(~H); A383, C(~O)(:C)(:C); A438, C(–C)(–N)(=C).

Interpretable molecular descriptors (1D&2D and ML_QD_) and fingerprints (MACCS, PubChem and Sub) were processed with machine learning algorithms to provide information on relevant structural features for the UV–Vis spectrum classification. MACCS, PubChem and Sub fingerprints are binary attributes that encode the presence or absence of a particular structural feature. The importance of attributes calculated by RF was inspected. Additional to RF, simple classification trees were grown to understand relationships between individual attributes and the potential photoreactivity of molecules. As expected, the trees are poorer predictive models than the more complex RF (Table 7) but are useful to analyze the importance of molecular fragments. The classification tree trained with PubChem fingerprints is shown in Fig. 2, and the trees obtained with the other attributes are in Figures S1–S4 of the Supplementary Material.

**Table 7:** Evaluation of classification trees based on interpretable fingerprints and molecular descriptors for the classification of UV–Vis absorption spectra of organic molecules in test set I.

Model	Q^a^	SP^b^	SE^c^	MCC^d^	TP^e^	TN^f^	FP^g^	FN^h^
1D&2D	0.73	0.71	0.76	0.47	380	353	146	119
ML_QD_	0.72	0.65	0.80	0.45	398	325	174	101
MACCS	0.71	0.67	0.75	0.42	373	335	164	126
PubChem	0.71	0.67	0.76	0.42	377	332	167	122
Sub	0.69	0.68	0.70	0.38	349	339	160	150

^a^Overall predictive accuracy for the training set in OOB estimation. ^b^Overall predictive accuracy (test set I). ^c^Specificity (test set I). ^d^Sensitivity (test set I). ^e^MCC, Matthews correlation coefficient (test set I). ^f^True Positives (test set I). ^g^True Negatives (test set I). ^h^False Positives (test set I). ^i^False Negatives (test set I).

In the tree obtained with PubChem fingerprints (Fig. 2) the first two rules use the presence of two conjugated C=C double bonds and the α,β-unsaturated carbonyl in fragment “O=C–C=C–C” as discriminant features. Additionally, the presence of aromatic fragments with nitrogen or oxygen substituents (“N–C:C:C–C” and “C(~O)(:C)(:C)”) were also used.

The features selected by the tree grown with MACCS fingerprints were similar and operated in similar ways. Features encoding the presence of aromaticity, double bonds connected to nitrogen atoms, as well as the absence of carbon atoms with at least two single bonds and at least two hydrogens were associated with the positive class.

Most of the inferred rules classify compounds as POS due to the *presence* of specific types of sub-structures, which agrees with the chemical knowledge that chromophores give rise to UV–Vis absorption. However, some rules associate the POS class with the absence of some aliphatic fragments. An example is the presence of a tertiary carbon atom; although it does not preclude the presence of chromophores in the molecule, a tertiary carbon atom is not involved in conjugation and is statistically associated with the negative class in our data set.

The 1D&2D molecular descriptors enabled a tree (in Figure S3) to infer two powerful rules based on the number of atoms in the largest pi system. They discriminate molecules with extended conjugation and fused bicyclic rings derived from e.g., naphthalene, indole, benzimidazole, benzoxazole, benzofuran, benzothiophene, or benzazepine. An additional rule is based on features of nitrogen atoms with two aromatic bonds.

Based exclusively on the estimated energies of the HOMO and LUMO orbitals, and their gap, a single rule was established that associates the POS class to GAP < 4.626 eV. Inspection of the database revealed that the molecules with the lowest value for this descriptor (~ 2 eV) include highly conjugated aromatic systems, such as tetracarboxdiimide derivatives, also corresponding to visible light absorption in the range between 500 and 700 nm with high MEC values (> 1000 Lmol^−1^ cm^−1^).

The ten most important attributes according to the *MeanDecreaseGini* parameter in the RF models are reported in Tables S1–S4 of the Supplementary Material for various fingerprints and descriptors. The most relevant attributes identified by the RF models are in line with those selected to build the trees, namely attributes accounting for the presence of aromaticity, unsaturation and conjugated systems. The importance of conjugation was highlighted by the selection of three Sub fingerprints that encode the presence of α,β-unsaturated carbonyl or carboxyl groups. The presence of tertiary carbon atoms is at the top ten in three models (MACCS, Sub and PubChem—Tables S1-S3). This feature was also used in the tree of Fig. 2 associated with the negative class. Although some of the 1D&2D descriptors have no straightforward interpretation, it is clear that different aspects of unsaturated systems are encoded by several of the most important attributes: number of atoms in the largest pi system, ratio of total conventional bond order with total path count, fraction of sp^3^ carbons to sp^2^ carbons, measure of relative unsaturation content, total number of bonds that have bond order greater than one. Furthermore, measures of global electronic features appear as highly relevant in positions 8 and 9.

### Analysis of outliers

**Figure 3:**
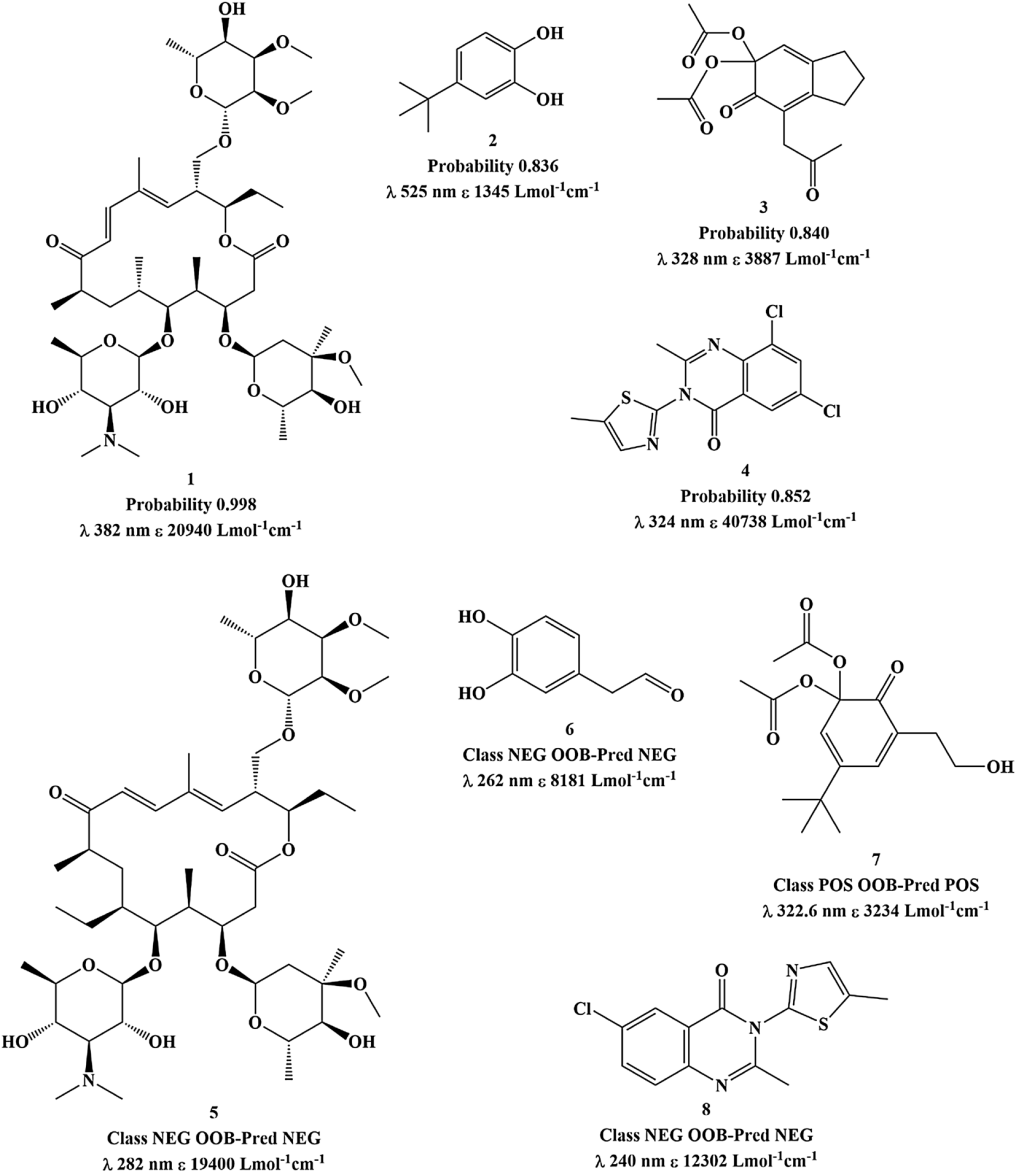
The chemical structures of four FN (**1**–**4**) predicted with high probability and their most similar training set counterpart structures (**5**–**8**). Experimental data is included as retrieved from the database.

The RF model trained with all RDKitMorgan fingerprints predicted the test set I with accuracy of 0.88 and MCC 0.76. The ROC curve of Fig. 1 illustrates the significance of the probabilities assigned by the RF models to the predictions. Among the 998 predicted molecules, the 15 FP and 18 FN with a probability higher than 0.8 were manually inspected to discover possible reasons for wrong predictions with high assigned probabilities. Most false negatives (12 out of 18 FN) correspond to molecules with peak wavelengths inside the photoreactivity window, but close to the lower endpoint of the interval (290–317 nm). Other 2 FN have a peak within the window but with a MEC value lower than 1500 Lmol^−1^ cm^−1^. The other 4 FN are compounds with peaks inside the window and high values of MEC reported in the database: a 16-membered macrolide with 3 deoxy sugar moieties attached and including a pi system (**1**)^34^, two cyclic compounds with conjugated systems (**2**^35^ and **3**^36^) and a quinazolinone connected to a thiazole (**4**)^37^, Fig. 3.

Three of these four predictions could be explained by similar molecules in the training set, which were assigned to the NEG class based on the experimental data. The structures **1**, **2**, **3** and **4** in Fig. 3 were subjected to a similarity search against the training set, using fingerprints and Tanimoto coefficients. Molecules **1**, **2** and **4** have a similar counterpart in the training set assigned to the NEG class and predicted as NEG in the OOB estimation: molecules **5**^34^ (Tanimoto coefficient of 0.99), **6**^38^ (Tanimoto coefficient of 0.70) and **8**^37^ (Tanimoto coefficient of 0.94), respectively. Compound **3** has a similarity of 0.83 with compound **7**^36^ of the training set, and both were assigned to the POS class based on the experimental data.

Although compound **5** is correctly assigned to the NEG class based on the experimental data, according to our definition of the POS and NEG classes (it has no maxima in the relevant window with MEC above the threshold), it represents in fact a borderline situation because UV–Vis absorption peaks are typically broad. Compound **5** has a peak at 282 nm with a MEC value of 19,400 Lmol^−1^ cm^−1^; it is therefore highly probable that the MEC value at 290 nm is higher than 1000 Lmol^−1^ cm^−1^ suggesting photoreactive potential. This example highlights a limitation of the models due to the nature of the experimental data here used (consisting in lists of UV–Vis absorption maxima): the *absence* (in the list) of a maximum within the relevant window *does not guarantee* that there is no absorptivity in the window with a MEC above the threshold. It may happen that the experimental spectrum did not cover the full window or the source publication reported only the highest peak(s), and there are often absorptivities in the window with a MEC above the threshold from bands whose maxima are outside the window. The similarity of compound **6** (NEG) to compound **2** may explain the prediction of the latter as negative; but inspection of the original source for compound **2** reveals that the reported UV–Vis data are for a metal complex and shall not be considered for structure **2**. This case illustrates how the method can be useful for the curation of experimental databases. Compound **8** may explain the false negative prediction for compound **4**; the inclusion of an additional chlorine substituent in the aromatic ring added a new absorption band at a higher wavelength^37^, and the ML model apparently did not learn that effect.

Concerning the 15 FP, it was observed that 4 molecules are among those in the NEG class with a peak at a wavelength only slightly below 290 nm (281–289.5 nm) and with a high MEC value. Other 3 FP have peaks at wavelengths between 269 and 277 nm with MEC values between 9000 and 28,183 Lmol^−1^ cm^−1^. Two FP have a peak with both the wavelength and MEC very near the thresholds (wavelength 295–306 nm, MEC 661–891 Lmol^−1^ cm^−1^). For the other 4 FP, all the MEC values listed in the database are between 3 and 5, which suggests they were originally reported in a different unit or as log(MEC)—confirmation was possible for at least one of them that a log(MEC) value was retrieved from the original literature as MEC and the compound exhibits indeed significant absorption within the 290–700 nm window. Finally, the other 2 FP arise from a situation similar to compounds **4** and **8**: a similarity search against the training set revealed that the inclusion/changing of a substituent in the aromatic system (in these cases to include methoxy and amino groups) is associated with the reporting of an absorption maxima at higher wavelengths.

### UV–Vis spectrum classification as a predictor of in vitro phototoxicity

For 43 molecules of test set II, additional data is available concerning the 3T3 NRU in vitro phototoxicity assay (PIV)^2^. The UV–Vis spectrum class was predicted for this subset with global accuracy 0.86, sensitivity 0.96 and specificity 0.69 (comparing to 0.89, 0.90 and 0.88, respectively, for the whole test set II—Table 6).

The RF output (UV–Vis spectrum class) was evaluated as a predictor of phototoxicity. Similarly, predictions of phototoxicity were also obtained using the lists of peaks and their MEC values available in the database of experimental data (“experimental spectrum class”). This enables to compare two approaches to the assessment of phototoxicity: (a) classification of the UV–Vis spectrum from lists of peaks available in the chemical literature, and (b) machine learning prediction of the spectrum class from the molecular structural formula. The confusion matrices are in Table 8.

**Table 8:** Confusion matrices relating RF-predicted and experimental UV–Vis spectrum class with the 3T3 NRU phototoxicity in vitro assay.

	Predicted UV–Vis spectrum class		Experimental UV–Vis spectrum class	
	POS	NEG	POS	NEG
Toxic	16	3	15	4
Non-toxic	15	9	12	12

The two confusion matrices are quite similar. The RF classification would correctly estimate 25 out of 43 molecules comparing to 27 using the experimental data. All the 12 non-toxic molecules assigned to the positive class in the experimental database were also predicted as positive by the RF model, and two toxic molecules were classified as negative both in the database and in the RF predictions. This suggests that a RF classification model for UV–Vis absorption features can assist in the estimation of in vitro toxicity similarly to experimental UV–Vis data. However, it must be emphasized that this study was not based on full spectra, but on lists of peaks extracted from the literature with their inherent incompleteness, and they certainly include noise.

The large number of non-toxic molecules assigned to the positive class is a limitation of UV–Vis spectra as a predictor of phototoxicity, because other characteristics of a chemical compound, beyond light absorption, are critical for phototoxicity, namely the ability to generate a reactive species. In any case, from the perspective of photosafety evaluation, high sensitivity is more important than specificity since molecules predicted as positive would be subjected to further experimental tests.

## Conclusion

The random forest algorithm, trained with 72,787 organic molecules represented by Morgan circular fingerprints, was able to classify molecules according to UV–Vis spectrum features related to photoreactive potential with accuracy up to 0.89, sensitivity of 0.90 and specificity of 0.88 for an independent test set of 998 molecules. The classes in the training and test sets were assigned based on data retrieved from the chemical literature, which consists of lists of absorption maxima with molar extinction coefficients.

Application of machine learning algorithms with interpretable molecular descriptors and fingerprints provided information on relevant structural features for the classification. The rules inferred by classification trees and the importance of attributes calculated by RF revealed that aromaticity, unsaturation, conjugation, and heteroatom substituents play an important role in discriminating between positive and negative classes.

Analysis of outliers (wrongly predicted molecules with high associated RF probability) highlighted three main situations: (a) absorption maxima with wavelengths near the lower endpoint of the established interval (290–700 nm) and/or MEC values close to the established threshold; (b) data noise, e.g., retrieval of log(MEC) value instead of MEC value; (c) insufficient learning of the impact of some heteroatom substituents on the absorption maxima.

The ML assignment of molecules to the positive class (related to photoreactive potential) was a predictor of a positive outcome of the 3T3 NRU phototoxicity in vitro assay with a sensitivity of 0.84 and specificity of 0.38 in a test set of 43 molecules. Comparable results were observed with the assignment based on the experimental data available for the same set (sensitivity 0.79 and specificity 0.5). The results illustrate the potential of machine learning algorithms for the classification of molecules according to the UV–Vis absorption spectrum, to assist in photosafety evaluation.

## Supplementary Information

Supplementary Information 1.

Supplementary Information 2.

## Data Availability

The dataset used in this study is provided by Elsevier Limited via the Reaxys database under license. The list of molecules is provided in the Supplementary Material in the SMILES notation with the corresponding Reaxys registry numbers; the Reaxys data is copyright (C) 2020 Elsevier Limited except certain content provided by third parties.
