# Supplementary material for: Machine learning prediction of UV–Vis spectra features of organic compounds related to photoreactive potential
Source: Sci Rep. 2021 Dec 9;11:23720. doi: 10.1038/s41598-021-03070-9 (PMC8660842; doi:10.1038/s41598-021-03070-9)
Supplement: Supplementary file 1 — Supplementary Information 1. [file 41598_2021_3070_MOESM1_ESM.pdf]

Machine Learning Prediction of UV-Vis Spectra Features of Organic Compounds Related to Photoreactive Potential

Rafael Mamede, Florbela Pereira, and João Aires-de-Sousa

SUPPLEMENTARY INFORMATION

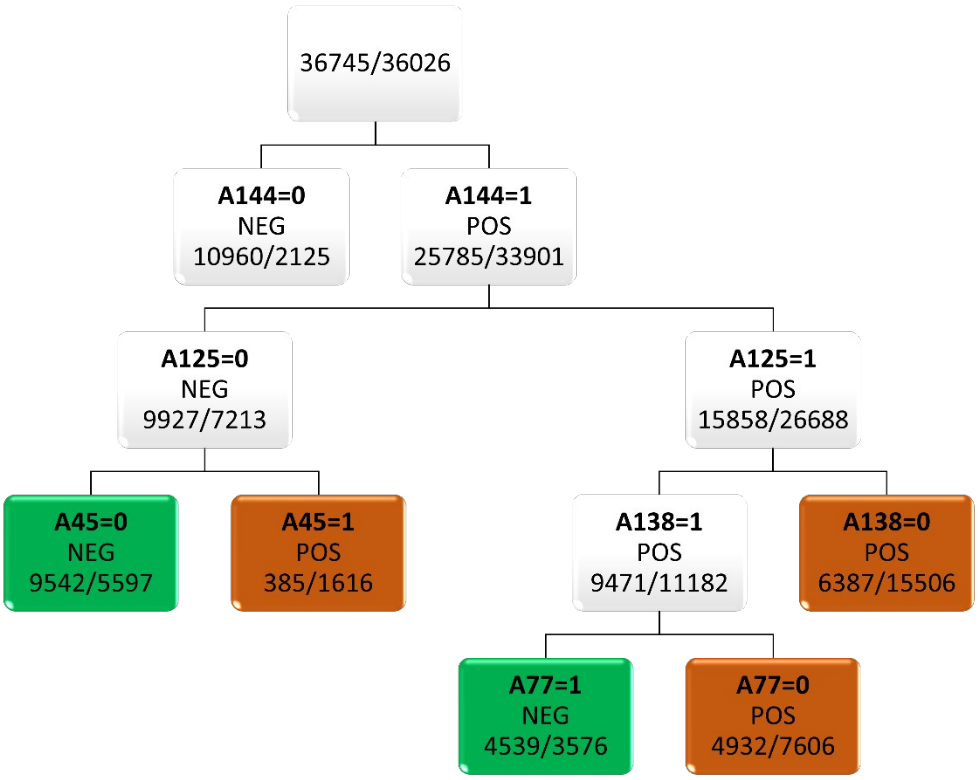

Figure S1. Classification tree based on MACCS fingerprints for the discrimination of molecules of the POS and NEG classes related photoreactive potential. A144, atom A is at an aromatic/nonaromatic boundary; A125, Aromatic ring > 1; A45, C=CN; A138, QCH2A (Q is an heteroatom); A77, NAN (A is any atom).

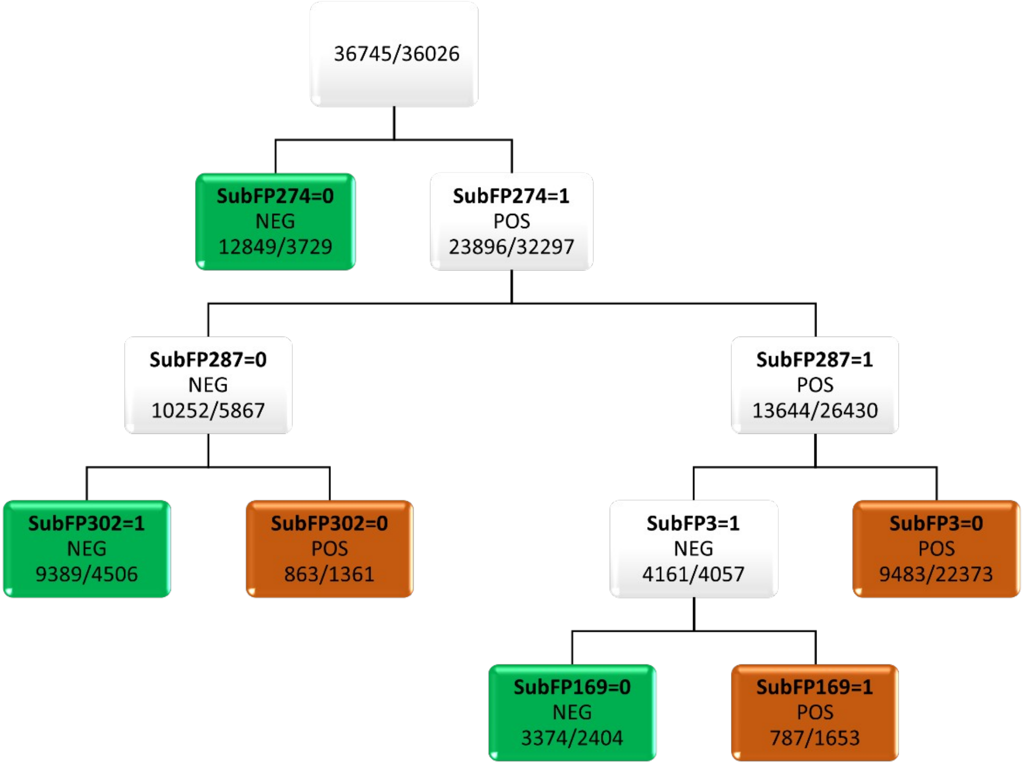

Figure S2. Classification tree based on Sub fingerprints for the discrimination of molecules of the POS and NEG classes related to photoreactive potential. SubFP274, Aromatic; SubFP287, Conjugated double bond; SubFP302, Rotatable bond; SubFP3, Tertiary carbon; and SubFP169, Phenol.

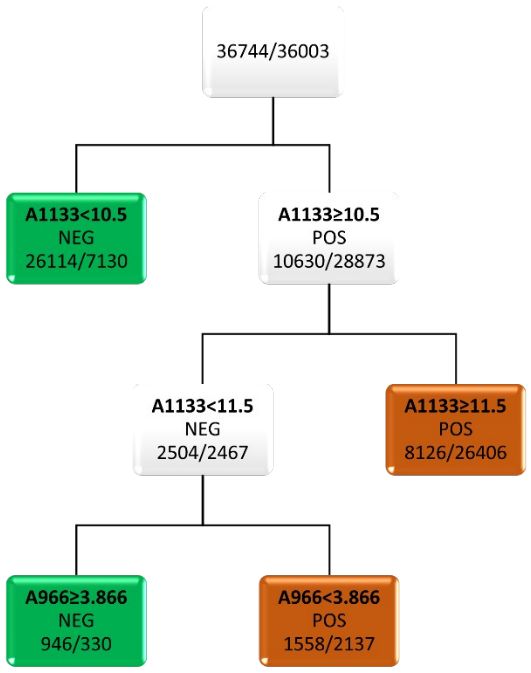

Figure S3. Classification tree based on 1D&2D descriptors for the discrimination of molecules of the POS and NEG classes related to photoreactive potential. A1133, nAtomP (number of atoms in the largest pi system); and A966, maxaaN (maximum E-state index of N atoms with two aromatic bonds).

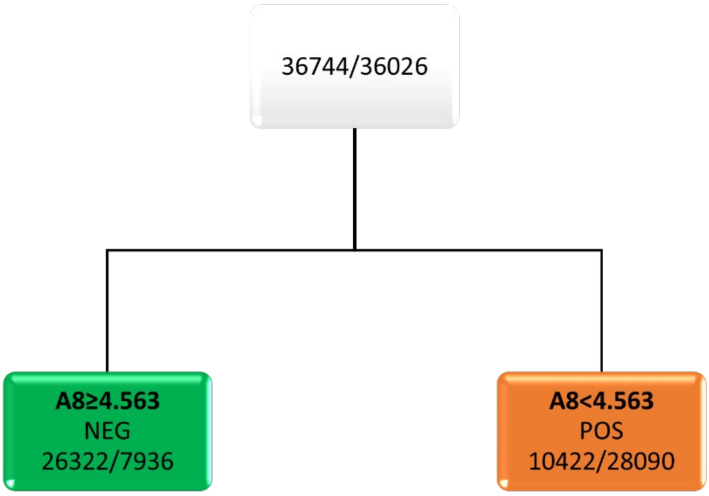

Figure S4. Classification tree based on ML_{QD} descriptors for the discrimination of molecules of the POS and NEG classes related to photoreactive potential. A8, GAP_CM.

Table S1. Top ten MACCS fingerprints listed in descending order of importance.

Code	MACCS FPs	Comments
A125	AROMATIC RING > 1	---
A113	Onot%A%A	
A105	A$A($A)$A	Atom, A, has more than two ring bonds
A144	Anot%A%Anot%A	Atom, A, is at an aromatic/nonaromatic boundary
A162	AROMATIC	---
A66	CC(C)(C)A	Atom, A, bonded to a tertiary carbon atom
A132	OACH2A	---
A129	ACH2AACH2A	---
A128	ACH2AAACH2A	---
A45	C=CN	---

Table S2. Top ten PubChem fingerprints listed in descending order of importance.

Code	Type	PubChem FPs	Comments
A672	SSP[1]	O=C-C=C-C	---
A665	SSP[1]	C-C=C-C=C	---
A336	SANN[2]	C(~C)(~C)(~C)(~H)	Tertiary carbon group
A554	SSP[1]	O=C-C=C	---
A565	SSP[1]	C=C-C=C	---
A438	DAN[3]	C(-C)(-N)(=C)	Methyl vinyl amine group
A340	SANN[2]	C(~C)(~C)(~H)(~O)	Secondary O-substituted group
A471	SSP[1]	C:C-C=C	α,β-unsaturated connected to aromatic bond
A433	DAN[3]	C(-C)(-C)(=O)	---
A258	ESSSR[4]	>= 2 aromatic rings	---

[1]Simple SMARTS patterns; [2]Simple atom nearest neighbors; [3]Detailed atom neighborhoods; [4]Rings in a canonic Extended Smallest Set of Smallest Rings.

Table S3. Top ten Sub fingerprints listed in descending order of importance.

Code	Sub FPs SMARTS	Description
SubFP274	a	Aromatic
SubFP3	[CX4H1]([#6])([#6])[#6]	Tertiary carbon
SubFP287	*=*[*]=,#,:[*]	Conjugated double bond
SubFP135	[#6X3](=[OX1])[#6X3]=,:[#6X3][#7,#8,#16,F,Cl,Br,I]	Vinylogous carbonyl or carboxyl derivative
SubFP137	[#6X3](=[OX1])[#6X3]=,:[#6X3][#6;!$(C=[O,N,S])]	Vinylogous ester
SubFP2	[CX4H2]([#6])[#6]	Secondary carbon
SubFP4	[CX4]([#6])([#6])([#6])[#6]	Quaternary carbon
SubFP169	[OX2H][c]	Phenol
SubFP12	[OX2H][CX4;!$(C([OX2H])[O,S,#7,#15])]	Alcohol
SubFP136	[#6X3](=[OX1])[#6X3]=,:[#6X3][$([OX2H]),$([OX1-])]	Vinylogous acid

Table S4. Top ten 1D&2D descriptors listed in descending order of importance.

Code	1D&2D descriptors	Description
A1133	nAtomP	Number of atoms in the largest pi system
A1183	R_TpiPCTPC	Ratio of total conventional bond order with total path count
A1086	HybRatio	Fraction of sp^3 carbons to sp^2 carbons
A1063	ETA_dBetaP	A measure of relative unsaturation content relative to molecular size
A1180	piPC9	Conventional bond order ID number[1] of order 9 (ln(1+x)
A451	nBondsM	Total number of bonds that have bond order greater than one (aromatic bonds have bond order 1.5).
A551	nwHBa	Count of E-States for weak hydrogen bond acceptors
A1057	ETA_BetaP	A measure of electronic features of the molecule relative to molecular size
A1061	ETA_BetaP_ns	A measure of electron-richness of the

		molecule relative to molecular size
A1182	TpiPC	Total conventional bond order (up to order 10) (ln(1+x))
[1]Accounts for multiple bonds in the molecule.		
